# To the Editors of the London Medical and Physical Journal

**Published:** 1818-09

**Authors:** 


*To the Editors of the London Medical and Physical Journal.*

GENTLEMEN,

THE following case, so extraordinary in its result, can be corroborated by the testimony of many credible witnesses, as well as by reference to the medical gentlemen who attended the patient; and, although not of the profession, I forward it to your valuable Journal, to give confidence to exertion, and to induce the afflicted never to despair, but to look forward with hope, even when disease shall have baffled all the efforts of medical art.

I am, Gentlemen, your obedient servant,

*August* 8, 1818. D. N. S.

---

MARY DIMMITT, ætat. 15, residing with her mother, a widow, with five children, at No. 3, Market-street, Borough-road, was attacked with a stiff neck and an enormous swelling under the right ear, on the 6th of May, 1817; which almost immediately dispersed without any application. On the 12th of the same month, after rising in perfect health, she was seized with a vertigo, and fell down stairs; from the effects of which she became deaf, and entirely lost her speech. She was admitted into Guy's Hospital on the 19th, and placed under the care of Mr. Lucas, surgeon, who ordered leeches to be immediately applied; and on the 20th, 21st, and 25th, she was alternately cupped, blooded, and blistered, without any apparent benefit.

May 30th, 10 P.M.—Her speech and hearing returned; and on the 2d of June she was dismissed the hospital, perfectly reinstated in health. In about a fortnight the vertigo returned, which was succeeded by five or six fits daily, and she entirely lost the use of her legs. Dr. M'Donnell, in the

Kent-road, attended her; but recommended her to be again placed in Guy's Hospital, into which she was admitted on the 5th of July, under the care of Dr. Marcet. Her fits continued, increasing in strength and number; and, from this period till the month of April 1818, when she was dismissed as incurable, she had undergone every experiment which that excellent establishment afforded: she had been repeatedly cupped, blistered, blooded; had setons and issues applied; the use of the cold-bath, &c.; and, in short, to use her own expression, "she suffered a martyrdom."

On her return home, the fits continued most violent, lasting frequently eight or ten hours: on her recovery from which she was perfectly unconscious of any pain, till the 26th of June, when she suffered excruciating agony from a swelling in her back. This was removed in five days, by the application of oils, &c. During all this long period of suffering she constantly used crutches.

On the 16th of July, she was again attacked in the usual manner; and, though the fit lasted only a quarter of an hour, she was sensible of much pain; but, on her recovery from which, she arose, and *walked across the room without any assistance whatever.* She has now thrown aside her crutches, and walks and runs as strongly as ever she did in her life.

July 28.—She had three fits, each lasting about a quarter of an hour: her right ankle swelled to the size of a pint basin, which dispersed by the application of oils, &c.

August 7.—On this day she has had twelve fits: she felt no other effect than a slight degree of langour and debility; and still retains the use of her limbs, as if she had never been deprived of them.

It is necessary only to add to this uncommon complication of severe sufferings, and to the extraordinary result, that during all this period she has continued very hearty, except during the time of the fits; and that her appetite not only never failed her, but it has exceeded the usual proportion of girls of her age. In the course of these fifteen months, she grew nearly thirteen inches in height.

